# Autonomic Dysregulation in Individuals With Psychiatric Disorders and Healthy Controls: Results From the CAP-MEM Observational Cohort

**DOI:** 10.1192/bjo.2022.186

**Published:** 2022-06-20

**Authors:** Tiago Costa, Sean Hill, Abigail Taylor, Anna Green, Francesca Black, Stuart Watson

**Affiliations:** 1 Newcastle University, Newcastle upon Tyne, United Kingdom; 2 Cumbria, Northumberland, Tyne and Wear NHS Foundation Trust, Newcastle upon Tyne, United Kingdom

## Abstract

**Aims:**

Psychiatric disorders are associated with fatigue and with impairment to a range of cognitive domains, including executive functioning, learning, memory and complex attention. Similar impairments are seen in autonomic nervous system (ANS) dysfunction. The aetipathogenic significance of this for psychiatric disorders is unknown. The main aim of the cap-mem study was to characterize the relationships between ANS and cognitive function in a sample of none-clinical controls and people with mental health, neurodevelopmental and neurodegenerative disorders. The potentially confounding role of medication was included within this analysis.

**Methods:**

The sample was recruited via secondary care mental health trusts. ANS function was assessed using self-report measures of ANS dysfunction symptoms (COMPASS-31) and fatigue (VAFS). Cognitive ability in various domains was measured using a validated, computerised assessment tool (THINC-IT). Psychiatric status and medication status were self-reported, and where possible, disorder severity measured using a rating scale (CGI-S).

**Results:**

Participants with depression had a significantly higher COMPASS-31 and VAFS scores (higher being more severe), with effect sizes being medium to large. Medication did not fully explain the associations observed. Overall, participants with mental health disorders, when compared to healthy controls, had significantly higher levels of cognitive impairment. Levels of ANS dysfunction significantly and positively correlated with cognitive impairment. The severity of the psychiatric disorder significantly correlated with both ANS dysfunction (p < 0.001) and cognitive impairment. These results were found across all cognitive tests (p < 0.05), other than reaction times in the N-back test, a measure of working memory.

**Conclusion:**

Our results show significant association between ANS dysfunction, psychiatric disorders and cognitive impairments. This is consistent with previously published data. There is now a need to understand the underlying mechanisms and the directionality of the associations. If these mechanisms are shared and relate to autonomic dysfunction, targeted treatments addressing this directly could be helpful with mental health disorders and associated burdensome symptoms, such as cognitive impairments and fatigue. This study is part of a wider project assessing cognitive ability and autonomic functioning in psychiatric populations, and investigating treatments that directly address autonomic dysfunction in psychiatric samples, such as non-invasive transauricular vagus nerve stimulation (taVNS).

